# Supplementary material for: Aromatic Characterisation of Moscato Giallo by GC-MS/MS and Validation of Stable Isotopic Ratio Analysis of the Major Volatile Compounds
Source: Biomolecules. 2024 Jun 16;14(6):710. doi: 10.3390/biom14060710 (PMC11201454; doi:10.3390/biom14060710)
Supplement: Supplementary file 1 [file biomolecules-14-00710-s001.zip › biomolecules-3041096-supplementary.pdf]

Table S1. Free aromatic compounds identified and quantified in the *Moscato Giallo* grape samples (LOQ = 1.0 μg/Kg).

	Free form [μg/Kg]																
	Sample 1	Sample 2	Sample 3	Sample 4	Sample 5	Sample 6	Sample 7	Sample 8	Sample 9	Sample 10	Sample 11	Sample 12	Sample 13	Sample 14	Sample 15	Sample 16	Sample 17
***trans*-furan linalool oxide**	168,5	123,6	107,9	191,5	88,8	55,6	147,6	168,7	197,7	132,8	53,8	124,2	143,1	95,9	84,3	77,6	91,9
***cis*-furan linalool oxide**	101,9	156,1	111,7	189,4	59,9	26,6	133,5	111,4	139,4	86,4	29,8	115,5	132,2	83,7	63,5	51,9	56,5
**linalool**	1306,2	994,0	1420,1	1771,5	910,2	411,5	1519,4	1249,2	1914,5	989,5	257,2	1056,6	1234,8	1284,9	962,6	694,8	811,0
**α-terpineol**	13,1	13,0	13,3	20,8	5,8	4,2	17,6	11,0	17,6	13,5	4,0	14,9	19,3	10,5	12,5	8,0	8,4
**terpinen-4-ol**	5,0	3,0	3,0	6,0	2,0	3,0	3,0	3,0	6,0	3,0	1,0	3,0	4,0	3,0	3,0	2,0	2,0
**β-citronellol**	6,9	10,4	4,4	13,4	7,8	2,9	7,1	6,4	7,3	5,7	2,9	6,0	2,7	8,1	3,6	2,9	5,3
**nerol**	17,0	10,9	10,8	10,7	6,3	14,1	9,0	8,5	8,6	13,9	19,4	14,6	8,0	8,0	7,0	12,0	10,9
**geraniol**	58,7	54,5	45,3	64,0	29,4	64,9	40,7	54,8	55,3	67,3	106,4	82,5	35,1	61,4	37,3	54,0	58,2
**geranic acid**	28,7	23,3	16,4	15,8	11,4	21,2	15,3	20,5	28,5	26,9	24,0	30,4	10,6	22,9	17,6	23,1	18,0
***trans*-rose oxide**	18,0	16,0	6,0	18,0	6,0	6,0	12,0	18,0	18,0	12,0	18,0	12,0	12,0	12,0	18,0	18,0	6,0
***cis*-rose oxide**	10,0	10,0	5,0	10,0	5,0	5,0	5,0	5,0	5,0	5,0	5,0	5,0	5,0	5,0	5,0	5,0	5,0
**limonene**	6,8	8,5	7,0	8,4	7,0	5,5	7,0	8,8	12,9	7,7	6,2	6,7	7,2	12,3	11,4	6,3	7,6
**myrcene**	12,6	10,7	13,8	16,5	13,2	12,6	16,2	15,1	24,9	16,1	10,9	10,1	12,3	21,5	20,0	9,6	14,3
**hotrienol**	26,0	7,0	28,2	48,2	12,5	7,9	18,7	13,6	30,4	19,9	13,7	28,3	27,9	21,4	12,4	18,1	8,7
***trans*-pyran linalool oxide**	480,0	548,5	298,1	514,9	304,5	167,5	352,3	511,5	561,5	346,0	147,2	346,9	324,7	337,0	320,3	257,3	248,8
***cis*-pyran linalool oxide**	137,8	197,3	133,9	179,5	107,3	70,5	159,8	143,4	149,9	121,4	79,5	148,3	142,3	107,3	110,9	110,4	125,2
**diendiol I**	576,0	212,1	281,5	408,6	150,0	180,5	361,2	298,7	742,6	360,7	134,6	388,7	436,7	259,3	231,6	172,3	249,8
**diendiol II**	424,7	929,4	246,4	433,5	187,0	86,9	207,5	389,9	325,5	268,8	157,9	252,8	290,3	288,7	269,3	236,9	144,1
***trans*-8-hydroxy linalool**	201,6	99,1	108,7	150,5	105,6	62,3	107,4	192,1	178,1	127,9	49,4	110,1	116,8	109,3	84,6	78,9	80,1
***cis*-8-hydroxy linalool**	29,2	43,7	23,9	40,2	29,9	20,8	27,2	19,6	44,2	20,8	16,7	24,6	16,8	29,6	21,3	33,9	31,9
**β-damascone**	< LOQ	< LOQ	< LOQ	< LOQ	< LOQ	< LOQ	< LOQ	< LOQ	< LOQ	< LOQ	< LOQ	< LOQ	< LOQ	< LOQ	< LOQ	< LOQ	< LOQ
**β-damascenone**	< LOQ	< LOQ	< LOQ	< LOQ	< LOQ	< LOQ	< LOQ	< LOQ	< LOQ	< LOQ	< LOQ	< LOQ	< LOQ	< LOQ	< LOQ	< LOQ	< LOQ
**α-pinene**	1,9	2,5	2,6	1,8	1,7	1,5	2,2	2,3	2,4	1,9	1,7	1,5	1,7	1,6	2,2	2,1	2,0
**α-ionone**	9,0	9,4	8,1	8,0	7,5	7,3	8,0	8,3	7,1	9,0	9,5	8,3	8,6	7,5	9,4	8,1	8,3
**α-ionol**	< LOQ	< LOQ	< LOQ	< LOQ	< LOQ	< LOQ	< LOQ	< LOQ	< LOQ	< LOQ	< LOQ	< LOQ	< LOQ	< LOQ	< LOQ	< LOQ	< LOQ
**1-hexanol**	159,0	129,2	83,3	54,8	100,3	101,9	81,1	67,3	97,3	90,9	58,1	77,7	78,1	123,6	113,3	53,7	77,8
***trans*-3-hexen-1-ol**	147,1	95,7	99,0	45,8	94,3	114,3	78,6	64,0	90,2	71,2	28,6	63,8	82,0	91,6	89,8	37,9	89,6
***cis*-3-hexen-1-ol**	117,4	116,8	87,4	74,9	92,9	99,7	84,4	61,1	88,2	95,0	78,8	96,8	85,0	116,8	98,3	65,0	85,1
**2-phenylethanol**	12,2	8,9	6,7	10,0	8,9	12,2	8,9	12,1	8,3	15,2	7,0	12,5	7,6	8,8	8,7	13,2	6,6
**benzyl alcohol**	15,0	16,1	11,1	16,4	11,9	19,1	13,0	15,3	19,2	18,0	12,3	15,3	9,8	12,7	12,5	14,7	8,9
**benzaldehyde**	2,0	2,2	2,8	6,5	1,9	1,9	2,4	1,7	2,3	1,9	1,8	2,2	2,3	1,7	2,0	1,7	1,4
**methyl salicylate**	< LOQ	< LOQ	< LOQ	< LOQ	< LOQ	< LOQ	< LOQ	< LOQ	< LOQ	< LOQ	< LOQ	< LOQ	< LOQ	< LOQ	< LOQ	< LOQ	< LOQ
**zingerone**	2,0	0,0	2,0	2,0	2,0	2,0	2,0	2,0	2,0	2,0	2,0	4,0	2,0	2,0	2,0	4,0	2,0
**guaiacol**	1,0	1,0	1,2	1,2	1,0	1,0	1,0	1,4	1,2	1,0	1,0	1,2	1,2	1,0	1,0	1,6	1,0

Table S2. Bound aromatic compounds identified and quantified in the *Moscato Giallo* grape samples (LOQ = 1.0 μg/Kg).

	**Bound form [μg/Kg]**																
	**Sample 1**	**Sample 2**	**Sample 3**	**Sample 4**	**Sample 5**	**Sample 6**	**Sample 7**	**Sample 8**	**Sample 9**	**Sample 10**	**Sample 11**	**Sample 12**	**Sample 13**	**Sample 14**	**Sample 15**	**Sample 16**	**Sample 17**
***trans*-furan linalool oxide**	91.4	139.8	77.7	89.4	46.2	53.8	72.8	107.6	122.4	64.0	51.8	70.5	66.9	92.0	76.5	53.7	49.0
***cis*-furan linalool oxide**	19.8	48.9	30.6	34.1	18.6	16.2	33.8	35.2	36.6	18.5	17.6	26.3	28.3	30.7	20.5	16.1	11.9
**linalool**	135.3	208.4	153.1	183.0	87.1	69.3	90.0	165.4	129.4	100.7	99.2	98.0	92.7	279.4	254.0	132.8	138.9
**α-terpineol**	30.1	27.0	33.0	32.2	15.7	23.2	31.0	33.1	30.1	24.7	27.8	22.7	25.4	41.4	31.5	21.4	18.1
**terpinen-4-ol**	8.5	6.5	10.5	7.0	5.0	6.5	7.0	6.5	6.5	4.5	8.5	5.0	7.5	3.0	4.5	5.0	5.5
**β-citronellol**	4.3	14.5	3.3	2.1	3.6	10.9	3.3	5.7	3.5	6.7	7.6	1.6	4.1	6.5	5.4	7.9	5.1
**nerol**	127.0	245.2	102.2	122.9	76.4	95.6	56.1	112.0	75.2	136.7	281.5	91.9	98.2	145.2	161.4	159.4	201.4
**geraniol**	411.9	907.1	349.4	541.0	326.8	411.7	223.6	394.5	251.3	457.0	1088.8	359.3	390.5	609.5	579.1	476.4	675.6
**geranic acid**	259.1	360.4	207.3	283.3	160.0	173.9	152.1	267.1	251.1	240.7	245.7	141.7	209.4	271.4	372.0	241.5	278.4
***trans*-rose oxide**	5.0	35.0	5.0	10.0	5.0	< LOQ	< LOQ	5.0	5.0	< LOQ	5.0	< LOQ	< LOQ	5.0	5.0	< LOQ	5.0
***cis*-rose oxide**	4.0	16.0	< LOQ	4.0	4.0	< LOQ	< LOQ	4.0	4.0	< LOQ	< LOQ	< LOQ	4.0	4.0	4.0	4.0	4.0
**limonene**	< LOQ	< LOQ	< LOQ	< LOQ	< LOQ	< LOQ	< LOQ	< LOQ	< LOQ	< LOQ	< LOQ	< LOQ	< LOQ	< LOQ	< LOQ	< LOQ	< LOQ
**myrcene**	< LOQ	< LOQ	< LOQ	< LOQ	< LOQ	< LOQ	< LOQ	< LOQ	< LOQ	< LOQ	< LOQ	< LOQ	< LOQ	< LOQ	< LOQ	< LOQ	< LOQ
**hotrienol**	39.0	13.5	31.7	32.0	11.2	17.5	18.1	21.7	13.5	14.3	25.4	14.1	21.4	9.9	7.7	18.0	19.4
***trans*-pyran linalool oxide**	28.5	20.7	17.7	14.3	13.6	17.4	18.5	24.5	25.2	13.4	17.3	10.2	24.8	22.8	21.8	17.0	17.1
***cis*-pyran linalool oxide**	27.0	31.8	20.4	40.7	23.7	20.0	29.6	30.0	37.9	25.1	31.3	24.2	29.4	32.5	35.3	53.9	26.6
**diendiol I**	505.1	314.7	265.9	372.2	164.3	215.4	160.6	468.0	469.6	304.6	193.3	253.5	315.6	370.6	293.4	239.7	242.7
**diendiol II**	16.6	83.8	18.0	33.0	8.8	9.2	11.9	25.6	24.8	7.2	17.7	14.8	8.5	33.0	43.3	16.3	17.3
***trans*-8-hydroxy linalool**	600.3	522.9	353.6	465.0	221.1	272.5	223.3	492.5	449.0	368.5	383.5	287.2	490.9	508.3	539.4	422.2	289.4
***cis*-8-hydroxy linalool**	204.8	830.9	168.0	235.8	109.3	125.3	100.9	168.1	149.9	158.2	230.6	145.1	200.5	243.5	249.3	197.8	201.6
**β-damascone**	80.5	76.3	90.4	84.0	81.3	77.2	74.5	96.3	71.5	77.2	82.3	91.2	77.6	74.2	83.2	88.3	79.4
**β-damascenone**	68.2	69.4	75.1	80.2	88.2	74.1	65.2	74.2	77.3	82.3	65.1	60.7	71.0	65.9	64.2	71.2	72.0
**α-pinene**	58.3	66.3	72.5	64.3	61.0	55.3	56.8	60.2	55.3	59.7	76.2	51.2	52.3	61.3	62.7	65.1	58.2
**α-ionone**	77.2	71.4	65.2	64.3	60.2	82.4	88.9	91.4	54.7	64.7	72.0	77.4	75.9	75.4	72.0	71.9	72.8
**α-ionol**	17.8	18.9	11.2	12.3	19.7	22.4	15.4	16.7	14.3	10.2	24.3	25.6	18.7	18.7	12.3	9.9	10.2
**1-hexanol**	13.7	9.6	17.6	21.6	12.6	10.5	11.9	17.9	11.5	22.3	19.6	17.2	15.0	25.3	25.7	22.6	23.6
***trans*-3-hexen-1-ol**	4.7	4.5	4.4	3.6	4.2	4.7	3.3	3.3	3.6	9.1	2.3	4.7	5.3	5.5	5.0	4.7	7.3
***cis*-3-hexen-1-ol**	1.3	1.5	1.5	1.5	1.1	2.1	1.0	1.1	1.4	2.5	1.5	1.6	1.8	1.4	1.4	1.4	1.7
**2-phenylethanol**	41.9	68.8	38.9	47.4	40.6	36.2	25.0	54.4	33.2	46.8	49.0	34.8	37.4	52.9	55.3	48.1	43.0
**benzyl alcohol**	29.5	60.4	22.5	29.3	26.7	35.9	18.8	40.8	31.8	33.9	37.5	19.9	23.4	41.1	41.0	34.5	29.4
**benzaldehyde**	4.5	6.0	4.0	10.5	5.0	5.5	4.0	5.5	4.5	7.0	4.0	4.0	5.0	4.0	4.0	4.0	4.5
**methyl salicylate**	2.1	2.5	2.4	2.9	2.8	2.7	2.3	2.5	2.8	3.1	3.4	2.2	2.0	2.5	1.7	1.6	1.5
**zingerone**	2.4	1.5	4.0	7.0	3.5	1.5	2.2	4.0	3.1	4.8	2.4	4.8	3.7	7.0	7.3	4.6	3.3
**guaiacol**	2.6	1.7	3.4	3.0	1.7	2.6	1.7	3.9	3.0	2.6	2.6	3.4	3.9	3.9	4.3	3.4	4.3

Table S3. Free aromatic compounds identified and quantified in the *Moscato Giallo* wine samples (LOQ = 1.0 μg/L).

	**Free form [μg/L]**																
	**Sample 1**	**Sample 2**	**Sample 3**	**Sample 4**	**Sample 5**	**Sample 6**	**Sample 7**	**Sample 8**	**Sample 9**	**Sample 10**	**Sample 11**	**Sample 12**	**Sample 13**	**Sample 14**	**Sample 15**	**Sample 16**	**Sample 17**
***trans*-furan linalool oxide**	150,3	114,1	93,8	182,7	77,9	65,3	135,3	155,8	181,1	129,3	45,3	107,0	130,3	84,8	82,9	83,8	84,8
***cis*-furan linalool oxide**	97,2	166,1	117,5	193,3	58,5	25,1	147,1	98,3	129,6	88,0	21,8	95,8	149,4	91,7	77,9	57,6	49,6
**linalool**	1364,7	999,0	1472,0	1824,6	877,0	483,7	1534,0	1165,3	1477,7	934,1	276,6	1154,1	1270,1	1179,1	966,3	628,1	790,9
**α-terpineol**	14,9	14,1	19,5	26,9	6,1	5,9	13,2	17,9	20,8	13,7	3,8	19,8	18,4	14,5	17,8	9,1	6,5
**terpinen-4-ol**	6,0	4,0	4,0	8,0	1,0	5,0	5,0	2,0	7,0	3,0	2,0	5,0	4,0	4,0	2,0	1,0	3,0
**β-citronellol**	7,0	10,0	4,9	18,2	8,5	3,4	5,1	6,7	7,5	6,0	3,2	6,5	2,9	8,9	7,1	2,3	6,0
**nerol**	15,5	11,2	11,5	11,9	7,8	13,0	8,4	8,9	12,6	17,4	21,9	13,9	9,6	6,8	5,9	13,4	12,6
**geraniol**	72,1	66,2	52,1	67,4	39,5	72,3	50,0	67,1	68,6	79,9	118,8	105,2	40,9	71,3	48,4	65,3	60,7
**geranic acid**	72,6	55,0	35,9	74,3	40,5	66,2	34,1	59,7	41,4	46,5	31,9	52,4	37,2	70,2	67,9	53,9	66,6
***trans*-rose oxide**	14,1	35,3	7,1	14,1	7,1	7,1	7,1	14,1	14,1	14,1	7,1	14,1	7,1	14,1	7,1	14,1	7,1
***cis*-rose oxide**	10,9	5,5	5,5	10,9	5,5	5,5	5,5	5,5	5,5	5,5	5,5	5,5	5,5	5,5	5,5	5,5	5,5
**limonene**	5,9	7,8	6,1	7,9	6,7	5,0	6,8	7,1	10,6	6,8	5,7	5,9	6,9	11,1	10,9	5,8	7,0
**myrcene**	11,1	8,6	12,2	12,1	12,0	12,9	11,4	14,2	22,2	12,0	9,5	9,8	11,5	14,4	13,3	8,3	12,8
**hotrienol**	6,7	1,4	3,8	1,8	1,5	10,0	5,8	8,6	8,7	5,1	7,6	5,7	7,8	2,5	5,8	9,2	2,1
***trans*-pyran linalool oxide**	479,1	531,3	319,3	493,3	353,4	170,4	319,6	550,4	584,0	388,0	149,8	326,8	286,2	356,0	304,9	265,3	240,5
***cis*-pyran linalool oxide**	148,8	180,7	110,6	106,3	94,5	48,9	142,9	126,0	109,0	96,6	48,5	164,8	169,4	103,9	71,9	78,3	111,4
**diendiol I**	908,0	507,9	426,0	715,0	234,4	352,7	487,3	595,0	954,3	610,4	384,5	651,8	676,2	578,0	426,4	415,7	414,0
**diendiol II**	412,5	901,4	255,1	466,2	173,0	85,4	211,9	409,6	310,7	242,4	195,6	245,0	299,3	265,6	295,4	213,4	144,3
***trans*-8-hydroxy linalool**	364,8	102,9	101,7	329,7	206,0	96,2	132,0	306,0	209,2	144,1	77,2	174,1	256,8	156,3	198,8	227,4	133,1
***cis*-8-hydroxy linalool**	112,4	327,5	64,6	148,7	94,8	41,4	27,7	51,0	95,4	79,2	63,3	71,0	38,8	107,9	97,1	101,5	87,2
**β-damascone**	25,6	26,4	30,2	35,4	42,0	39,1	25,6	26,4	24,4	31,1	32,3	34,1	29,8	27,6	26,4	30,1	27,4
**β-damascenone**	40,2	45,6	35,6	37,9	42,3	41,2	35,8	36,0	37,9	39,1	42,1	40,0	41,1	47,2	35,2	36,3	44,3
**α-pinene**	36,1	39,4	30,0	35,4	26,7	29,8	34,0	33,6	37,2	35,6	24,9	39,7	41,2	45,2	35,9	36,1	35,2
**α-ionone**	34,0	41,2	37,2	39,7	45,2	36,1	35,6	35,9	26,7	36,1	35,2	39,4	29,8	24,9	35,4	33,6	30,0
**α-ionol**	7,2	6,5	7,8	8,1	6,2	4,2	6,2	5,4	7,2	7,1	6,9	8,9	8,7	8,8	7,9	7,0	7,2
**1-hexanol**	487,1	516,2	321,2	687,1	543,3	329,2	321,2	357,2	433,4	371,1	232,8	383,2	613,6	618,0	502,3	391,9	403,0
***trans*-3-hexen-1-ol**	123,5	92,6	92,1	43,0	83,6	91,7	72,0	62,2	82,2	62,1	22,4	62,4	74,7	83,2	82,5	32,2	82,9
***cis*-3-hexen-1-ol**	109,4	95,4	74,3	66,4	88,8	87,8	76,0	53,2	64,1	84,3	65,1	89,4	77,1	98,7	77,9	41,7	66,7
**2-phenylethanol**	1,8	2,9	3,5	9,0	1,8	2,7	3,0	2,3	3,8	2,3	2,0	3,0	3,9	2,7	2,3	1,8	2,0
**benzyl alcohol**	2,3	2,5	3,4	3,6	5,4	2,1	3,1	2,0	3,3	4,2	4,4	5,2	2,1	2,0	1,9	3,0	2,8
**benzaldehyde**	1,2	1,3	2,7	6,2	2,3	1,0	1,0	2,0	6,6	3,5	1,6	1,2	1,0	1,2	3,9	0,4	0,4
**methyl salicylate**	3,2	2,9	3,4	5,2	1,5	2,5	3,2	2,8	2,4	3,1	3,5	4,6	2,1	2,8	3,0	3,3	3,5
**zingerone**	150,3	114,1	93,8	182,7	77,9	65,3	135,3	155,8	181,1	129,3	45,3	107,0	130,3	84,8	82,9	83,8	84,8
**guaiacol**	97,2	166,1	117,5	193,3	58,5	25,1	147,1	98,3	129,6	88,0	21,8	95,8	149,4	91,7	77,9	57,6	49,6

Table S4. Bound aromatic compounds identified and quantified in the *Moscato Giallo* wine samples (LOQ = 1.0 µg/L).

	**Bound form [µg/L]**																
	**Sample 1**	**Sample 2**	**Sample 3**	**Sample 4**	**Sample 5**	**Sample 6**	**Sample 7**	**Sample 8**	**Sample 9**	**Sample 10**	**Sample 11**	**Sample 12**	**Sample 13**	**Sample 14**	**Sample 15**	**Sample 16**	**Sample 17**
***trans*-furan linalool oxide**	78,9	124,3	61,2	71,4	32,3	41,0	68,9	90,5	104,6	45,7	32,8	59,2	50,5	86,5	67,2	40,1	39,9
***cis*-furan linalool oxide**	13,8	34,1	22,4	28,5	13,6	8,7	22,7	32,8	24,3	17,4	15,7	23,2	12,8	22,4	18,5	15,4	9,5
**linalool**	119,6	196,7	136,6	163,6	77,9	53,6	71,3	150,5	117,4	72,0	85,0	69,2	80,4	214,1	216,2	112,6	126,9
**α-terpineol**	28,1	19,4	27,3	21,0	12,9	21,0	27,3	23,9	23,4	15,0	25,1	12,8	22,1	35,6	25,3	20,8	12,3
**terpinen-4-ol**	6,6	6,1	7,7	6,6	4,4	5,5	6,1	5,5	5,5	3,3	8,3	4,4	5,5	2,2	3,3	3,9	2,2
**β-citronellol**	4,1	12,1	2,3	1,5	2,3	10,4	2,0	4,1	3,1	5,3	6,1	1,4	2,4	5,3	4,6	6,9	3,1
**nerol**	104,3	237,8	93,4	83,8	67,4	65,8	42,3	96,0	68,1	127,0	213,3	62,2	53,5	117,1	153,6	140,4	198,1
**geraniol**	328,7	829,0	308,1	506,9	281,0	349,3	206,1	354,0	215,6	445,0	912,1	346,9	385,1	517,2	567,4	308,4	592,2
**geranic acid**	207,2	316,5	180,4	218,4	137,8	132,2	122,5	211,1	232,1	212,0	180,0	126,6	177,0	200,5	313,6	213,0	237,2
***trans*-rose oxide**	< LOQ	< LOQ	< LOQ	< LOQ	< LOQ	< LOQ	< LOQ	< LOQ	< LOQ	< LOQ	< LOQ	< LOQ	< LOQ	< LOQ	< LOQ	< LOQ	< LOQ
***cis*-rose oxide**	< LOQ	< LOQ	< LOQ	< LOQ	< LOQ	< LOQ	< LOQ	< LOQ	< LOQ	< LOQ	< LOQ	< LOQ	< LOQ	< LOQ	< LOQ	< LOQ	< LOQ
**limonene**	< LOQ	< LOQ	< LOQ	< LOQ	< LOQ	< LOQ	< LOQ	< LOQ	< LOQ	< LOQ	< LOQ	< LOQ	< LOQ	< LOQ	< LOQ	< LOQ	< LOQ
**myrcene**	< LOQ	< LOQ	< LOQ	< LOQ	< LOQ	< LOQ	< LOQ	< LOQ	< LOQ	< LOQ	< LOQ	< LOQ	< LOQ	< LOQ	< LOQ	< LOQ	< LOQ
**hotrienol**	4,4	6,3	5,2	2,2	2,1	3,5	3,4	6,9	5,0	4,4	3,3	3,7	5,6	3,5	2,9	3,4	3,3
***trans*-pyran linalool oxide**	15,7	16,5	12,4	7,7	10,9	12,3	12,9	17,0	15,3	4,3	13,6	5,2	21,2	15,4	17,4	11,5	16,5
***cis*-pyran linalool oxide**	12,8	15,6	14,5	9,9	5,8	5,0	5,6	18,2	14,7	12,9	10,7	7,5	14,4	16,0	10,4	9,7	8,8
**diendiol I**	40,3	32,2	36,5	33,3	18,2	10,6	13,9	53,6	52,2	25,5	19,0	18,3	15,6	49,6	43,7	23,5	22,3
**diendiol II**	11,0	74,6	12,3	20,4	2,1	8,8	5,0	14,9	15,2	6,0	7,1	4,8	6,6	28,4	36,1	12,6	7,8
***trans*-8-hydroxy linalool**	400,9	511,0	337,2	281,2	139,7	193,4	216,0	404,9	366,2	234,5	261,3	213,4	363,8	421,1	353,1	298,6	209,7
***cis*-8-hydroxy linalool**	122,7	547,5	145,5	124,1	70,0	65,7	103,0	140,7	98,7	82,5	122,1	98,5	172,2	157,7	135,4	114,8	122,4
**β-damascone**	45,0	47,4	55,3	52,9	48,1	49,0	44,8	46,8	53,2	40,6	66,2	68,8	41,9	43,0	38,9	47,4	54,4
**β-damascenone**	32,3	24,4	26,4	26,4	35,4	27,6	30,1	26,4	30,2	27,4	39,1	42,0	34,1	25,6	29,8	31,1	25,6
**α-pinene**	16,8	20,8	24,6	16,7	23,9	21,3	33,9	29,9	29,6	23,7	29,2	20,8	27,2	31,9	19,6	24,2	40,2
**α-ionone**	54,4	43,0	55,3	34,8	52,9	47,4	68,8	40,6	43,2	36,2	38,9	49,0	46,8	48,1	25,0	37,4	41,9
**α-ionol**	12,3	8,2	11,4	7,7	7,6	9,7	11,2	1,2	5,5	9,7	8,5	9,2	12,9	8,8	9,9	8,4	10,2
**1-hexanol**	8,5	4,1	9,5	17,5	7,8	5,4	6,5	11,9	6,8	13,1	7,9	9,9	9,5	14,6	17	15,5	15
***trans*-3-hexen-1-ol**	4,2	3,0	2,5	2,1	1,7	2,3	2,1	2,3	2,3	6,7	1,3	4,8	4,6	5,3	4,4	2,9	1,0
***cis*-3-hexen-1-ol**	1,3	2,3	2,3	2,2	1,4	2,2	1,1	1,8	2,2	3,8	1,4	2,0	2,2	1,8	2,3	2,2	2,5
**2-phenylethanol**	1,0	2,3	2,6	1,3	1,3	3,0	2,3	2,6	1,3	2,6	2,3	1,0	2,6	2,3	3,0	1,0	1,3
**benzyl alcohol**	< LOQ	< LOQ	< LOQ	< LOQ	< LOQ	< LOQ	< LOQ	< LOQ	< LOQ	< LOQ	< LOQ	< LOQ	< LOQ	< LOQ	< LOQ	< LOQ	< LOQ
**benzaldehyde**	1,7	0,7	1,7	2,9	1,4	1,0	1,0	1,7	1,0	2,0	1,2	2,9	2,4	5,1	3,7	3,2	2,2
**methyl salicylate**	2,5	2,6	3,4	3,9	3,4	4,1	2,5	2,4	2,7	3,5	3,3	3,6	2,5	1,9	3,2	3,0	3,3
**zingerone**	78,9	124,3	61,2	71,4	32,3	41,0	68,9	90,5	104,6	45,7	32,8	59,2	50,5	86,5	67,2	40,1	39,9
**guaiacol**	13,8	34,1	22,4	28,5	13,6	8,7	22,7	32,8	24,3	17,4	15,7	23,2	12,8	22,4	18,5	15,4	9,5

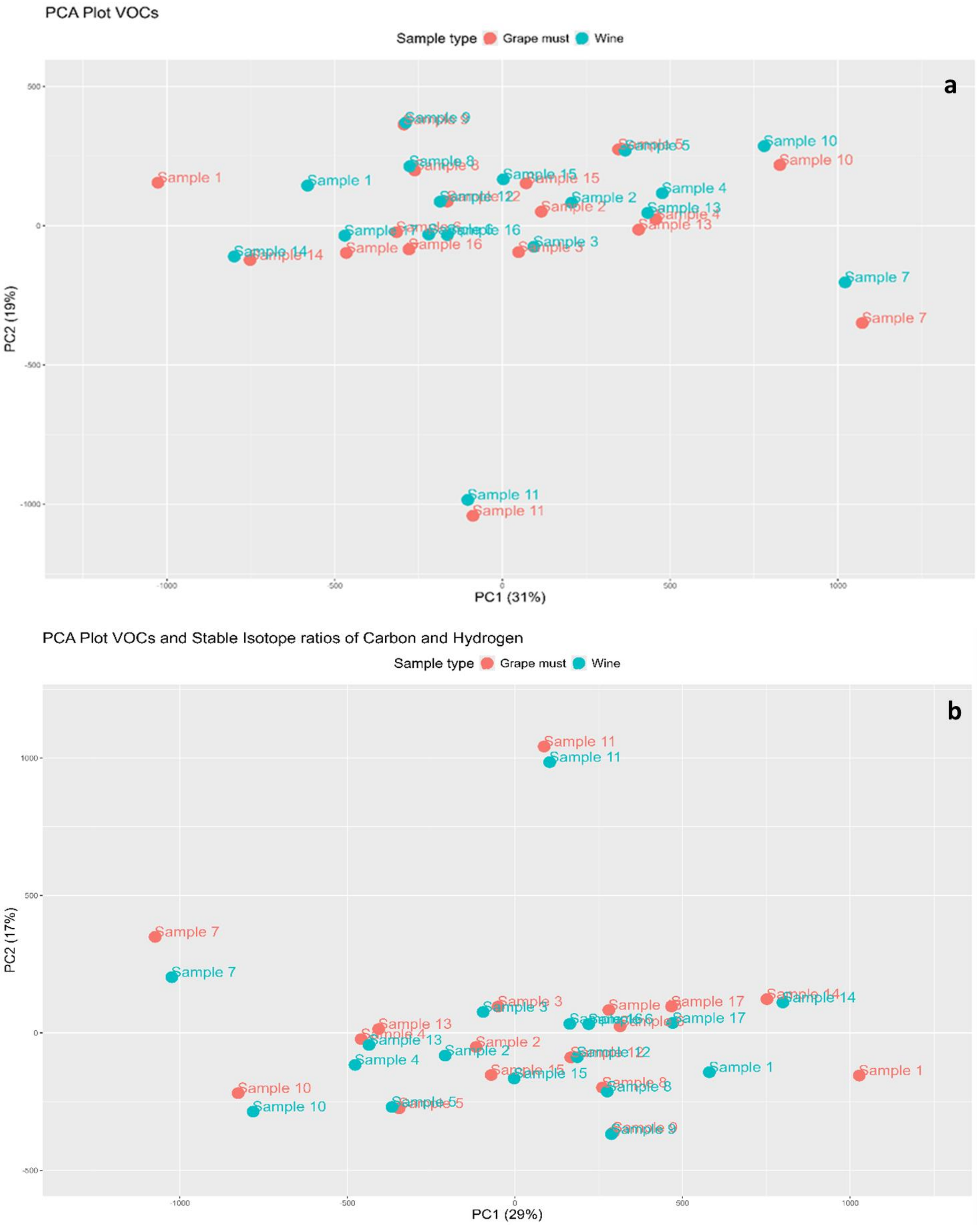
PCA Plot VOCs
Sample type
Grape must
Wine
a
PC2 (19%)
PC1 (31%)
500
0
-500
-1000
-1000
-500
0
500
1000
Sample 1
Sample 7
Sample 10
Sample 11
Sample 14
Sample 16
Sample 2
Sample 3
Sample 4
Sample 13
Sample 15
PCA Plot VOCs and Stable Isotope ratios of Carbon and Hydrogen
Sample type
Grape must
Wine
b
PC2 (17%)
PC1 (29%)
1000
500
0
-500
-1000
-500
0
500
1000
Sample 11
Sample 7
Sample 3
Sample 13
Sample 17
Sample 14
Sample 2
Sample 4
Sample 1
Sample 10
Sample 15
Sample 9

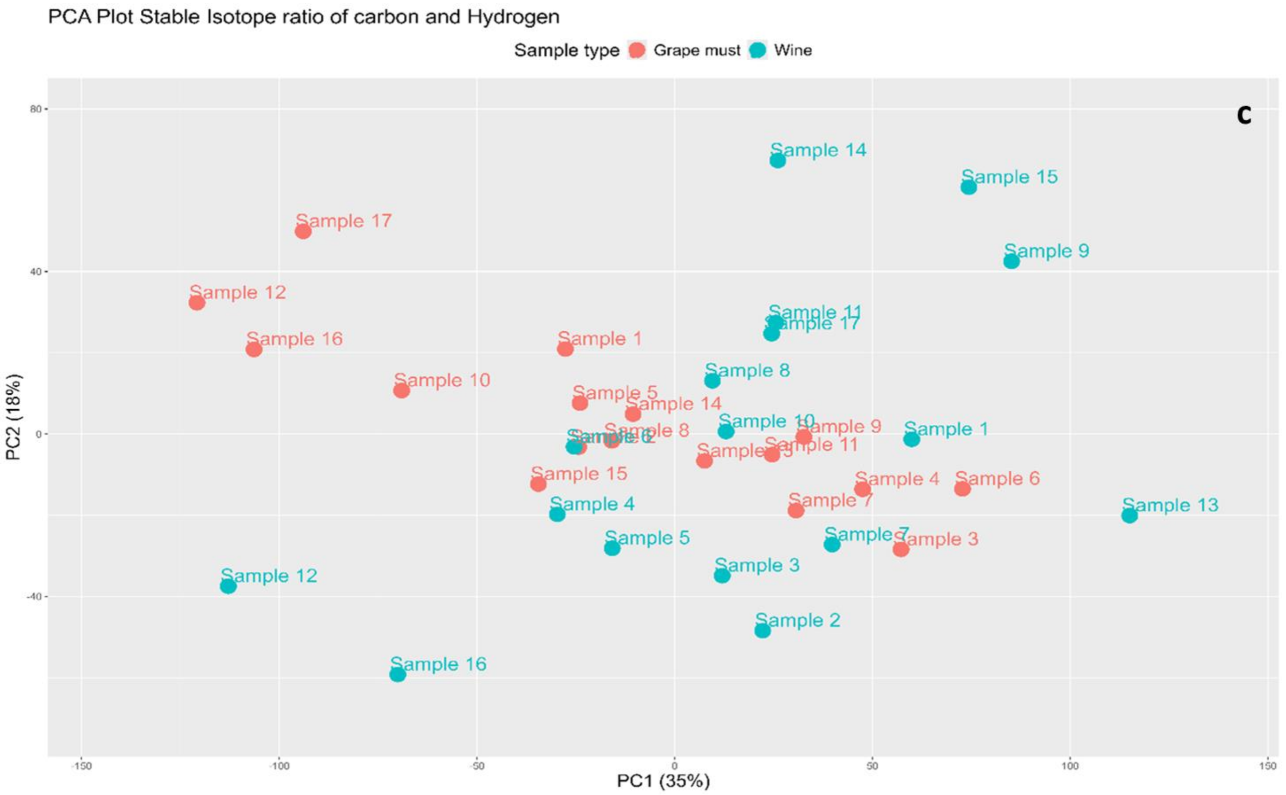

Figure S1. PCA score plots: a) VOCs; b) VOCs and compound-specific δ^{13}C and δ^{2}H; c) compound-specific δ^{13}C and δ^{2}H.
